# Rate Coefficient and Branching Ratio for the Formation of Criegee Intermediate S*yn*-/A*nti*-CH_3_CHOO from CH_3_CHI + O_2_ and the Self-Reaction of S*yn*-/A*nti*-CH_3_CHOO Determined with Simultaneous IR/UV Probes

**DOI:** 10.1021/acs.jpca.4c06588

**Published:** 2024-10-20

**Authors:** Tang-Yu Kao, Chen-An Chung, Yuan-Pern Lee

**Affiliations:** † Department of Applied Chemistry and Institute of Molecular Science, National Yang Ming Chiao Tung University, Hsinchu 300093, Taiwan; ‡ Center for Emergent Functional Matter Science, National Yang Ming Chiao Tung University, Hsinchu 300093, Taiwan

## Abstract

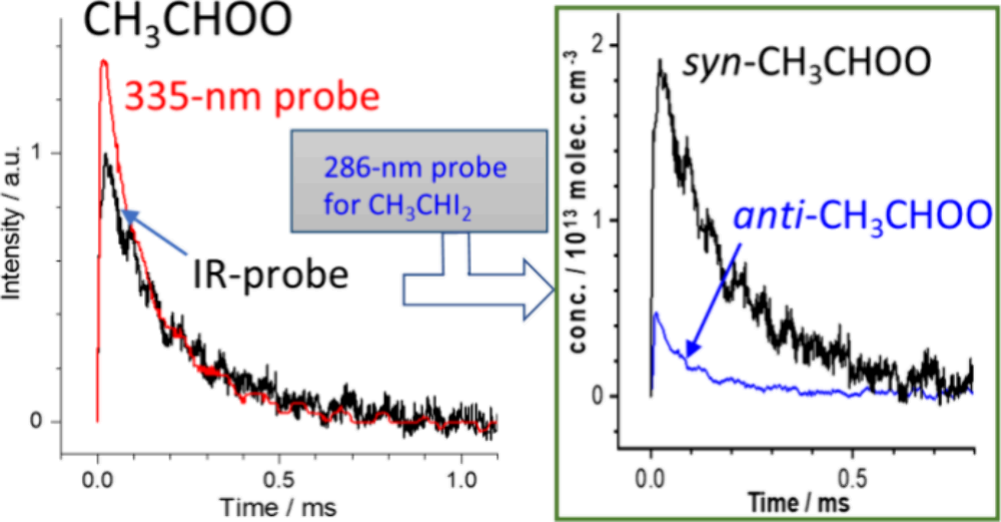

A flow reactor coupled with a light-emitting diode at 286 nm, an infrared quantum-cascade laser near 11 μm, and an ultraviolet laser at 335 nm was implemented to probe the precursor CH_3_CHI_2_, *syn*-CH_3_CHOO, and *anti*/*syn*-CH_3_CHOO, respectively, in the reaction of CH_3_CHI + O_2_. The branching between *syn*- and *anti*-CH_3_CHOO was determined to be ≈80:20 from two methods. The concentration temporal profiles of *anti*-CH_3_CHOO, derived on comparison of infrared and ultraviolet profiles, yielded the rate coefficient for the self-reaction of *anti*-CH_3_CHOO, *k*_self_^anti^ = (6 ± 2) × 10^–10^ cm^3^ molecule^–1^ s^–1^, ∼4 times the corresponding value, *k*_self_^syn^ = (1.4 ± 0.3) × 10^–10^ cm^3^ molecule^–1^ s^–1^, for *syn*-CH_3_CHOO; the rate coefficient for the cross-reaction between *syn*-CH_3_CHOO and *anti*-CH_3_CHOO was estimated to be (2.1 ± 0.6) × 10^–10^ cm^3^ molecule^–1^ s^–1^. With determined concentrations of *syn*-CH_3_CHOO and self-reaction rate coefficients, the rate coefficient for the formation of CH_3_CHOO from CH_3_CHI + O_2_ was determined to be *k*_form_ = (3.8 ± 0.7) × 10^–12^ cm^3^ molecule^–1^ s^–1^ at 298 K, ∼45% of previous reports.

## 1 Introduction

Carbonyl oxides, also known as Criegee intermediates, were produced from the ozonolysis of unsaturated organic compounds; they play critical roles in the production of OH under dim-light conditions^1−6^ and the formation of acids and secondary organic aerosols (SOA) in the atmosphere.^2,3,7,8^ In laboratory studies, small Criegee intermediates were produced via photolysis of diiodoalkanes in the presence of O_2_.^9−11^ This method made the direct probe of the carbonyl oxides using various spectral methods feasible, and consequently led to valuable insights into their reactivities, kinetics, and reaction mechanisms.^3,11−17^ For larger Criegee intermediates, various conformers might exist and the conformation-specific chemistry plays important roles in atmospheric chemistry.^14^

The two-carbon Criegee intermediates, acetaldehyde oxide (CH_3_CHOO), were generated in the atmosphere through the reactions of ozone with 2-alkenes. Taatjes et al. employed the reaction of CH_3_CHI + O_2_ to synthesize CH_3_CHOO and detect it with multiplex photoionization mass spectrometry (MPIMS).^10^ These authors were able to distinguish the two conformers, *syn*-CH_3_CHOO and *anti*-CH_3_CHOO, by their distinct ionization thresholds, ∼9.4 and 9.3 eV, respectively. According to quantum-chemical calculations, *syn*-CH_3_CHOO is more stable than *anti*-CH_3_CHOO by 11–16 kJ mol^–1^.^18−20^ A significant barrier of ∼160 kJ mol^–1^ prevents the interconversion between these conformers at ambient temperature.^20,21^

Sheps et al. reported broad and overlapping ultraviolet (UV) absorption bands of *syn*-CH_3_CHOO and *anti*-CH_3_CHOO, with peak positions near 323 and 360 nm, respectively.^22^ These spectra were derived by making use of the distinct reactivities of these two conformers toward H_2_O and SO_2_; *anti*-CH_3_CHOO has much greater reactivity than *syn*-CH_3_CHOO, so that it was depleted on adding excess H_2_O or SO_2_. Our group measured transient IR spectra of CH_3_CHOO with a step-scan Fourier-transform infrared (FTIR) spectrometer.^23^ Because the infrared (IR) spectra of *syn*-CH_3_CHOO and *anti*-CH_3_CHOO are similar and the practical spectral resolution was limited to ∼0.5 cm^–1^, most bands of *syn*- and *anti*-CH_3_CHOO were overlapped, so that they could only be distinguished through simulation of rotational contours according to high-level full-dimensional quantum-chemical calculations. To better resolute the detailed spectral information, our group later utilized a quantum-cascade laser coupled with a Herriott absorption cell to record the OO-stretching band of CH_3_CHOO in region 880–932 cm^–1^ with resolution 0.0015 cm^–1^; although spectral analysis was challenging, several lines solely due to *syn*-CH_3_CHOO were identified when CH_3_OH was added to the system to deplete *anti*-CH_3_CHOO via its rapid reaction.^24^ Liu et al. reported several absorption bands in region 5600–6100 cm^–1^ that are associated with overtone and combination bands of *syn*-CH_3_CHOO by detecting with laser-induced fluorescence the OH radicals that were produced upon IR activation of cold CH_3_CHOO;^25,26^ no band of *anti*-CH_3_CHOO could be identified with this method.

CH_3_CHOO serves as a prototype for understanding the conformation-specific reactivity.^14,15,27^ The *syn*-CH_3_CHOO, with the terminal O atom interacting with two H atoms of the methyl group, decomposes to OH radicals through the 1,4-hydrogen transfer, leading to a more rapid unimolecular decomposition than *anti*-CH_3_CHOO.^16,25,26,28^ Previous studies demonstrated that *anti*-CH_3_CHOO is significantly more reactive toward H_2_O and SO_2_ than *syn*-CH_3_CHOO.^10,22,29−33^ Additionally, *syn*- and *anti*-CH_3_CHOO show distinct differences in bimolecular rate coefficients in reactions with NO_2_,^10,34^ CH_3_OH,^35−37^ HC(O)OH,^38^ CH_3_C(O)OH,^38^ amino alcohol,^39,40^ and dimethylamine (DMA).^41^

To perform accurate conformation-specific kinetic analysis, it is crucial to monitor *syn*- and *anti*-CH_3_CHOO distinctively. Using the multiplex photoionization mass spectrometry (MPIMS), Taatjes et al. differentiated these two conformers by using ionization at 10.5 and 9.35 eV for the detection of *syn*- and *anti*-CH_3_CHOO respectively. According to these authors, at 10.5 eV, *syn*-CH_3_CHOO accounts for ∼80% of CH_3_CHOO.^10^ The UV absorption bands of *syn*- and *anti*-CH_3_CHOO, with maxima near 323 and 360 nm, respectively, are significantly overlapped.^22^ To probe the UV absorption of *anti*-CH_3_CHOO with a negligibly small contribution of *syn*-CH_3_CHOO at wavelength greater than 400 nm is possible,^22^ but the absorption of *anti*-CH_3_CHOO is small and IO might interfere in this region; below 360 nm, both *syn*- and *anti*-CH_3_CHOO have significant absorption. Consequently, previous kinetic measurements on reactions of *syn*-/*anti*-CH_3_CHOO with atmospheric species using UV absorption either monitored the rapid decay and ascribed it to *anti*-CH_3_CHOO or fitted the decay with two decay components and ascribed them to *anti*- and *syn*-CH_3_CHOO, respectively. Liu et al. reported that OH radicals were produced solely from the unimolecular decomposition of *syn*-CH_3_CHOO;^42^ they probed OH by laser-induced fluorescence to investigate the kinetics of *syn*-CH_3_CHOO + HCl. To the best of our knowledge, distinct probes of *syn*-CH_3_CHOO and *anti*-CH_3_CHOO simultaneously for kinetic studies have not been reported.

In this work, we set up a new IR/UV dual-probe multipass absorption system to determine the branching between *syn*- and *anti*-CH_3_CHOO and conduct kinetic measurements for the formation of CH_3_CHOO and the self-reaction of *syn*- and *anti*-CH_3_CHOO. This system combines the advantages of high-resolution IR absorption of a quantum-cascade lasers (QCL) near 11 μm, which can selectively detect *syn*-CH_3_CHOO, and UV absorption at 335 nm, which can detect both *syn*- and *anti*-CH_3_CHOO and quantify precisely their concentrations. Furthermore, absorption of light at 286 nm from a light-emitting diode (LED) quantifies the concentration of the CH_3_CHI_2_ precursor, hence the CH_3_CHI radical produced upon irradiation at 248 nm. With these probes, we were able to derive precisely the branching between *syn*- and *anti*-CH_3_CHOO, rate coefficients for the self-reactions of *syn*- and *anti*-CH_3_CHOO, and the rate coefficient for the formation of CH_3_CHOO from CH_3_CHI + O_2_.

## 2 Methods

**Figure 1:**
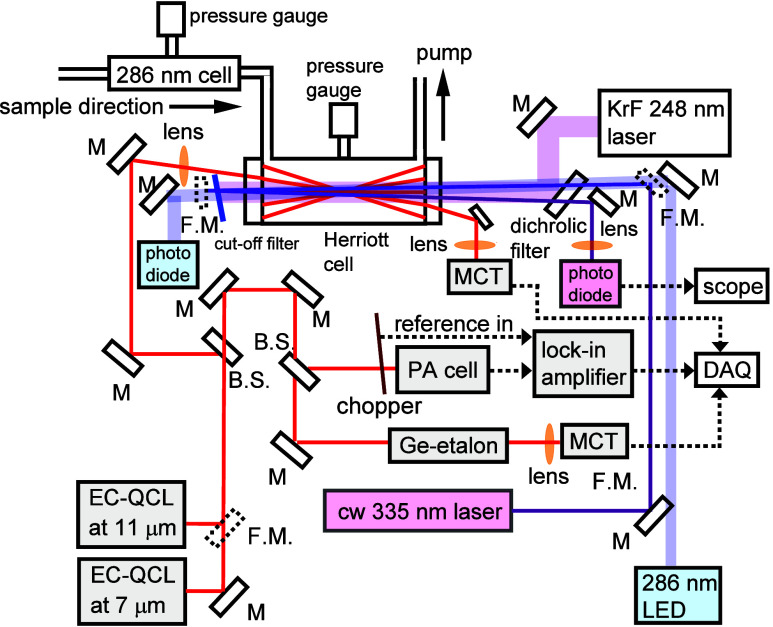
UV/IR dual-probe experimental setup. We utilized external-cavity quantum-cascade lasers (EC-QCL) and a 335 nm laser coupled with a Herriott multipass absorption cell (effective path length ∼19 m) to record time-resolved IR spectra of *syn*-CH_3_CHOO and UV spectra of *syn*-/*anti*-CH_3_CHOO. Additional light-emitting diode at 286 nm quantifies the concentration of the precursor CH_3_CHI_2_. B.S.: beam splitter; M: mirror; F. M.: flip mirror; PA: photoacoustic cell; MCT: HgCdTe detector; DAQ: data acquisition system.

The experimental setup is depicted in Figure 1. The flow reactor is equipped with a pair of Herriott mirrors (diameter 51 mm, radius of curvature 40 cm) at each end with a separation of 75.4 cm and an effective path length of ∼18.9 m; the central part (2.5 cm in diameter) of both mirrors was removed to allow passage of the photolysis beam at 248 nm from a KrF excimer laser (Coherent, Compex Pro 102F, max power ∼300 mJ pulse^–1^); CaF_2_ windows were installed at both ends of the reactor. *syn*- and *anti*-CH_3_CHOO were produced upon irradiation at 248 nm of a flowing mixture of CH_3_CHI_2_ and O_2_ at 5–9 Torr and 298 K. Typically, the photolysis laser was operated at 7.0 Hz with a beam size ∼23 × 15 mm^2^ and energy ∼140 mJ pulse^–1^ before entering the reactor.

A Peltier-cooled continuous wave (*cw*) external-cavity quantum-cascade laser (QCL), operating in a mode-hop-free mode near 11 μm (Daylight Solutions, 41112-MHF, resolution ∼ 0.002 cm^–1^), was coupled to the Herriott absorption cell to record time-resolved IR absorption spectra in region 880–932 cm^–1^. Before entering the reactor, this output was split to have two additional beams to pass a germanium etalon (FSR = 0.025 cm^–1^) and a photoacoustic reference cell for wavelength calibration. An absorption path ∼12.4 m was estimated to be overlapped with the photolysis beam. After passing through the Herriot cell, the QCL beam was detected with a photovoltaic HgCdTe detector (Kolmar, KMPV13-1-J2, cooled to 77 K; typically, both ac-coupled and dc-coupled outputs were recorded with a data-acquisition board (Spectrum, M2p 5962-x4, 16 bit, up to 125 × 10^6^ samples s^–1^). For taking a spectrum, the step size for scanning the wavenumber of the QCL was approximately 0.002 cm^–1^, with a probed duration of 2.1 s after wavelength tuning. During this probed duration, the photolysis UV laser was triggered 15 times at 7 Hz and the signal was averaged over 15 measurements at each wavelength; the sampling rate was typically set at 2–5 M samples s^–1^. For kinetic measurements, to avoid the shift of wavelengths, instead of setting the QCL at a specific wavenumber, we scanned the spectrum near 883 cm^–1^ and integrated the band in spectral regions 883.105–883.135 and 883.148–883.185 cm^–1^_._ A minimal concentration of *syn*-CH_3_CHOO is ∼5 × 10^12^ molecules cm^–3^ for practical kinetic measurements.

The system also incorporates two additional UV probes. A light-emitting diode (LED) at 286 ± 7 nm (with a nearly parallel beam of size ∼2.3 cm^2^ after passing through several lenses) was injected into the reactor via a dichroic mirror (Semrock, Di01-R266-25X36), which reflects the photolysis light at 248 nm and passes light at 286 nm. This probe beam was completely within the photolysis volume and was focused onto a Si detector (Thorlabs, PDA10A-EC, 200–1100 nm) via an elliptic mirror; the absorption length was estimated to be 87 cm. This LED probe determined the variation of [CH_3_CHI_2_] upon photolysis, hence the initial concentration of CH_3_CHI [CH_3_CHI]_0_, according to an absorption cross-section of CH_3_CHI_2_ at 286 nm (3.6 × 10^–18^ cm^2^).^43^

The second UV probe was a diode-pumped solid-state laser operating in a *cw* mode at 335 nm (CNI Laser, UV–F-335, 10 mW, ∼0.6 mm in diameter). To increase the absorption length, the probe beam was reflected once by an external mirror placed outside the Herriott cell and detected with a Si detector (Thorlabs, PDA10A-EC, 200–1100 nm); the effective absorption length inside the reactor was 174 cm. The absorption cross sections of *syn*- and *anti*-CH_3_CHOO at 335 nm were estimated to be 11.3 × 10^–18^ and 8.5 × 10^–18^ cm^2^ molecule^–1^, respectively, according to the figure of Sheps et al.^22^

In a typical experiment, we passed O_2_ through liquid CH_3_CHI_2_ before entering the reactor. A 51 cm absorption cell with light at 286 ± 7 nm from a LED and a Si-photodiode detector (Thorlabs, PDA10A-EC, 200–1100 nm) was installed upstream of the reactor to measure the partial pressure of CH_3_CHI_2_. The partial pressures of CH_3_CHI_2_, O_2_, and He in the reactor were evaluated from the flow rate of each gas steam, controlled by a mass flow controller (MKS). The concentration of CH_3_CHI_2_ in the reactor was also double-checked with the 286 nm LED to ensure consistency. CH_3_CHI_2_ (96–98%, Orgchem Tech.), O_2_ (99.99%, Chiah-Lung), and He (99.9995%, Chiah-Lung) were used as received.

## 3 Results and Discussion

### 3.1 Branching of CH_3_CHI + O_2_ → CH_3_CHOO

**Figure 2:**
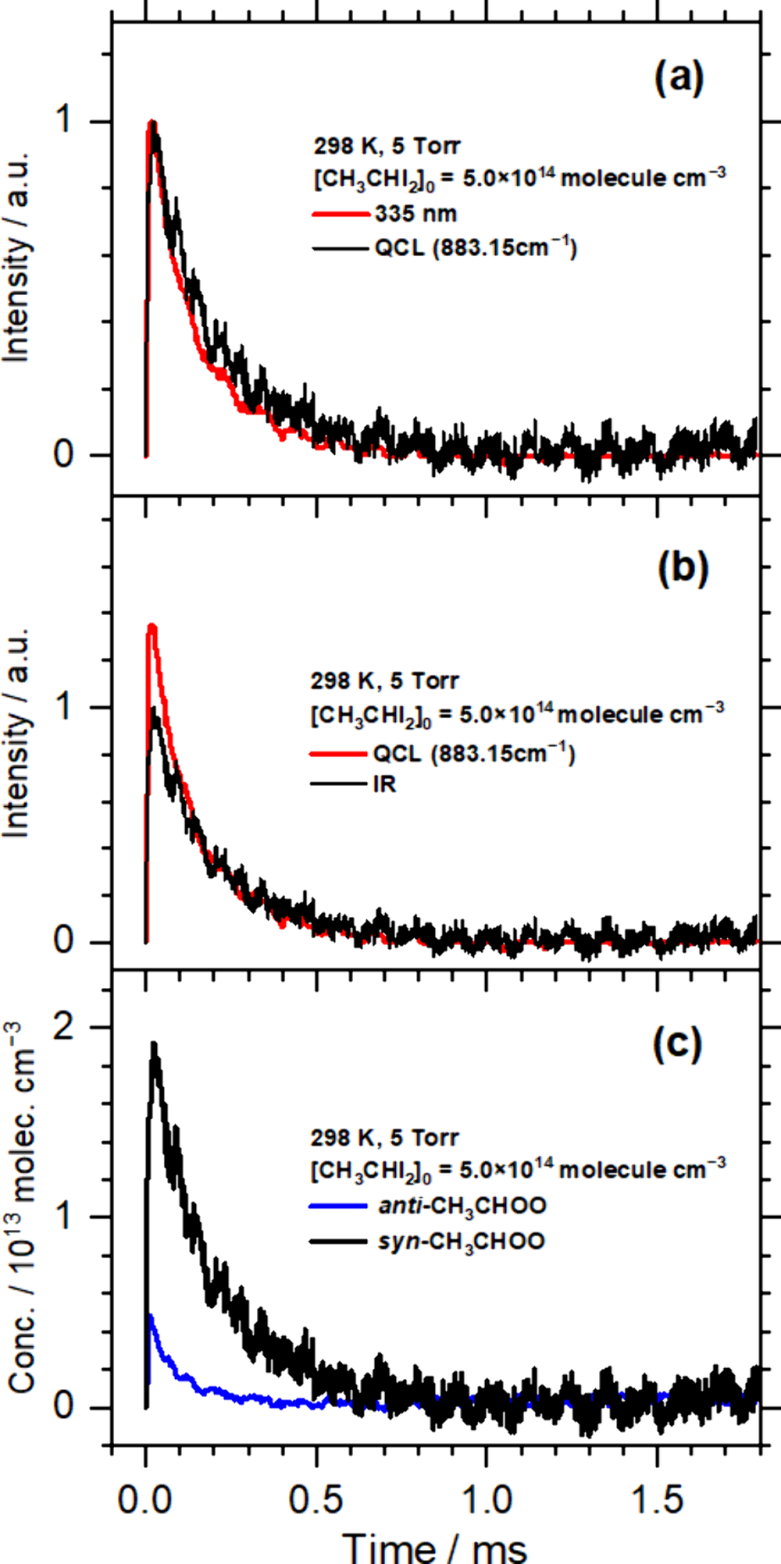
Derivation of temporal profiles of *syn*- and *anti*-CH_3_CHOO. IR (∼883.15 cm^–1^, black) and UV (335 nm, red) absorption temporal profiles are compared in (a), with maxima normalized, and (b), with the slow decay matched. IR absorption is due to only *syn*-CH_3_CHOO, whereas UV absorption is due to *syn*- and *anti*-CH_3_CHOO. (c) Comparison of temporal profiles of *syn*-CH_3_CHOO (black) and *anti*-CH_3_CHOO (blue), derived by subtracting the black curve from the red curve in (b) and conversion to concentration.

Following the report of Luo et al.,^24^ we probed the absorption band of *syn*-CH_3_CHOO in regions 883.105–883.135 and 883.148–883.185 cm^–1^. The UV light at 335 nm was absorbed by both *syn*- and *anti*-CH_3_CHOO.^22^ Hence, the temporal profiles of IR and UV absorption measured simultaneously are expected to differ in absorption of *anti*-CH_3_CHOO, as demonstrated in Figure 2. The UV (red) and IR (black) profiles normalized to the intensity maxima are shown in Figure 2a; the decay of the red trace is slightly more rapid than that of the black trace. Figure 2b compares the two traces with the slow decay (after ∼0.3 ms) matched; the slow decay component is presumably due to only *syn*-CH_3_CHOO. The UV absorption profile (red) contains an additional component, as compared with the IR profile (black, *syn*-CH_3_CHOO). This component corresponds to the UV absorption of *anti*-CH_3_CHOO. After conversion to concentrations, the temporal profiles of *syn*-CH_3_CHOO (black) and *anti*-CH_3_CHOO (blue) are shown in Figure 2c. This procedure demonstrates that we could probe both *syn*- and *anti*-CH_3_CHOO simultaneously by comparison of the UV and IR probes.

A detailed discussion on how we derived the concentrations of [CH_3_CHI]_0_ is presented in Section SA, Supporting Information. [CH_3_CHI]_0_ was determined from Δ[CH_3_CHI_2_], probed by the 286 nm LED (Figure S1). We employed two methods to measure the branching between *syn*- and *anti*-CH_3_CHOO. In Method A, we used the UV absorption at 335 nm and the LED absorption at 286 nm, and the reported σ_syn_ and σ_anti_ at 335 nm to derive the branching ratio. The branching ratios for the three channels of CH_3_CHI + O_2_
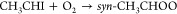
1a
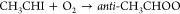
1b
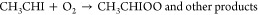
1care *a*: *b*: *c*, in which *a* + *b* + *c* = 1. Because O_2_ was in excess to react with all CH_3_CHI,
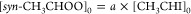
2a
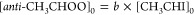
2b

The absorbance at 335 nm, *A*_335_^0^, right after the reaction of CH_3_CHI with O_2_ is hence
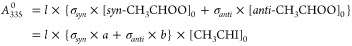
3in which σ_syn_ and σ_anti_ are absorption cross sections of *syn*-CH_3_CHOO and *anti*-CH_3_CHOO at 335 nm, and *l* (= 174 cm) is the absorption path length. We employed σ_syn_ and σ_anti_ as ∼11.3 × 10^–18^ and 8.5 × 10^–18^ cm^2^ molecule^–1^, respectively, at 335 nm, according to the figure of Sheps et al.^22^ However, according to the figure in a recent paper by Lade et al., σ_syn_ and σ_anti_ are ∼9.5 × 10^–18^ and 5.2 × 10^–18^ cm^2^ molecule^–1^, respectively, at 335 nm.^44^ The deviations in σ_syn_ and σ_anti_ are ∼16 and ∼39%, respectively; the σ_anti_/σ_syn_ value at 335 nm is 0.75 from Sheps et al. and 0.55 from Lade et al. On the other hand, the σ_anti_/σ_syn_ value at 335 nm is 0.86 from Lin et al.^31^ We hence adapted the value 0.75 from Sheps et al. and consider a possible error of 27%.

Hence,
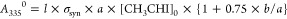
4

At low pressure, *a* + *b* = 0.86 ± 0.11 (in 2 Torr of Helium) according to Howes et al.^29^ This value is expected to be similar for pressure below 10 Torr. Hence, eq 4 can be deduced to
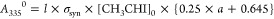
5
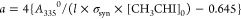
6

Because the reaction of CH_3_CHI + O_2_ typically takes 3–7 μs to reach 90% completion, the [CH_3_CHOO]_0_, hence *A*_335_^0^, could not be determined directly upon irradiation at 248 nm. We assumed that the key loss of CH_3_CHOO is due to its self-reaction, so we plotted 1/*A*_335_ versus time and extrapolated the value to *t* = 0 to obtain *A*_335_^0^, as shown in Figure S2. In Table S1, we listed the experimental conditions and [CH_3_CHI]_0_, 1/*A*_335_^0^, and *a* derived using eq 6 (Method A) in each experiment. Satisfactorily consistent results of *a* values were found; the average value gives *a* = 0.69 ± 0.06, in which the error limit represents one standard deviation in fitting. The averaged *a* value implies *b* = 0.17 ± 0.06 and *a*/(*a* + *b*) = 0.80 ± 0.07; the branching ratio for *syn*-CH_3_CHOO:*anti*-CH_3_CHOO is hence *a*:*b* = (80 ± 7):(20 ± 7).

This ratio of [*syn*-CH_3_CHOO]_0_:[*anti*-CH_3_CHOO]_0_ can be cross-checked by comparison of temporal profiles of UV absorption at 335 nm and IR absorption in regions 883.105–883.135 and 883.148–883.185 cm^–1^ (Method B). By subtracting the scaled IR profile from the UV profile, shown in Figure 2b, we derived the UV absorption of *anti*-CH_3_CHOO; the UV absorption of *syn*-CH_3_CHOO was consequently derived. By taking into account of σ_syn_ and σ_anti_, we derived temporal profiles of [*syn*-CH_3_CHOO] and [*anti*-CH_3_CHOO], as shown in Figure 2c.

The branching ratio [*syn*-CH_3_CHOO]_0_/{[*syn*-CH_3_CHOO]_0_ + [*anti*-CH_3_CHOO]_0_} = *a*/(*a* + *b*) derived from Method B are also listed in Table S1 for comparison with those derived from Method A. The average value *a*/(*a* + *b*) = 0.74 ± 0.09, derived from Method B, gives *a* = 0.64 ± 0.08, consistent with the value *a* = 0.69 ± 0.06 determined from *A*_335_^0^ and Δ[CH_3_CHI_2_] (Method A); both methods employed the reported cross sections of *syn*- and *anti*-CH_3_CHOO.

Considering the error in the measurements of [CH_3_CHI]_0_ (8%), *A*_335_^0^ (3%), UV cross-section (∼16%) of *syn*-CH_3_CHOO, the error in σ_anti_/σ_syn_ (∼27%), the error in *a* + *b* (∼13% from 0.86 ± 0.11), and the fitting error of *b* (35%), we estimated the overall uncertainty in the branching of *anti*-CH_3_CHOO (*b*) to be ±50%. We hence report *b* = 0.17 ± 0.09. The branching between *syn*-CH_3_CHOO:*anti*-CH_3_CHOO is hence (80 ± 10):(20 ± 10). This ratio is between the ratio ∼70:30 (10 Torr of He at 293 K) reported by Sheps et al.^22^ and that (∼90:10) reported by Taatjes et al.;^10^ these two values are within our error limit.

### 3.2 Rate Coefficients for the Self-Reactions of S*yn*- and A*nti*-CH_3_CHOO

A preliminary analysis of the self-reactions of *syn*-CH_3_CHOO and *anti*-CH_3_CHOO,
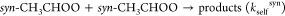
7
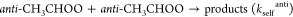
8by assuming no cross-reactions between *syn*-CH_3_CHOO and CH_3_CHOO,
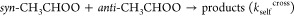
9is presented in Section SB, Supporting Information; some representative plots of [*syn-*CH_3_CHOO]^−1^ vs *t* (time) and [*anti-*CH_3_CHOO]^−1^ vs *t* (time) are shown in Figure S3. A summary of experimental conditions and the fitted results are shown in Table S2; a statistical distribution of these measurements of *k*_self_^syn^ and *k*_self_^anti^ appear in Figure S4. The average values of the model fit (Model A, listed in Table S3) gave *k*_self_^syn^ = (1.5 ± 0.2) × 10^–10^ cm^3^ molecule^–1^ s^–1^ and *k*_self_^anti^ = (10.2 ± 1.5) × 10^–10^ cm^3^ molecule^–1^ s^–1^. These values are the upper limits of *k*_self_^syn^ and *k*_self_^anti^; *k*_self_^anti^ is expected to be decreased more significantly than *k*_self_^syn^ after considering the cross-reaction because [*anti*-CH_3_CHOO]_0_ is much smaller than [*syn*-CH_3_CHOO]_0_.

**Figure 3:**
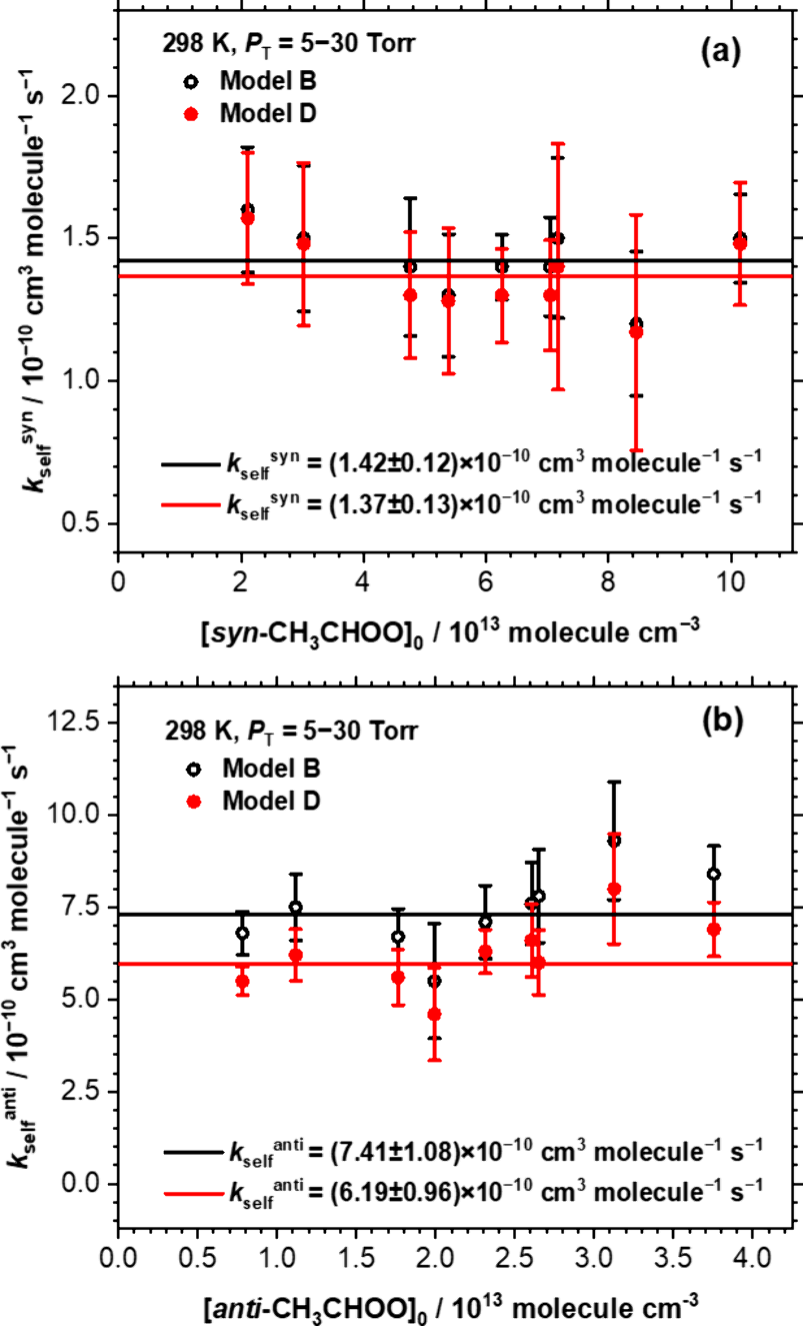
Comparison of *k*_self_^syn^ and *k*_self_^anti^ derived from Model B and Model D. (a) *k*_self_^syn^, with [*syn-*CH_3_CHOO]_0_ = (2.1–10.2) × 10^13^ molecules cm^–3^, *P*_T_ = 5.0–30.0 Torr, and *T* = 298 K. (b) *k*_self_^anti^, with [*anti-*CH_3_CHOO]_0_ = (0.8–3.8) × 10^13^ molecules cm^–3^, *P*_T_ = 5.0–30.0 Torr, and *T* = 298 K. Model B: open symbols, *k*_self_^cross^ = 1.5 × 10^–10^ cm^3^ molecule^–1^ s^–1^; Model D: solid symbols, *k*_self_^cross^ = 2.1 × 10^–10^ cm^3^ molecule^–1^ s^–1^.

With limited information, to derive accurately all three rate coefficients *k*_self_^syn^, *k*_self_^anti^, and *k*_self_^cross^ from our model fitting is challenging; we hence used a range of *k*_self_^cross^ values to derive *k*_self_^syn^ and *k*_self_^anti^. When we added reaction 9 in the model listed in Table S3, and used *k*_self_^cross^ = 1.5 × 10^–10^ cm^3^ molecule^–1^ s^–1^, the value of *k*_self_^syn^ derived without considering *k*_self_^cross^, as the lower limit of *k*_self_^cross^ (Model B), the derived *k*_self_^syn^ and *k*_self_^anti^ are listed in Table S4 and shown as open symbols in Figure 3. The average values are *k*_self_^syn^ = (1.42 ± 0.12) × 10^–10^ cm^3^ molecule^–1^ s^–1^ and *k*_self_^anti^ = (7.4 ± 1.1) × 10^–10^ cm^3^ molecule^–1^ s^–1^. For the upper limit of *k*_self_^cross^, we consider that the loss of *anti*-CH_3_CHOO was due to reaction with either *syn*-CH_3_CHOO or *anti*-CH_3_CHOO and assume that *k*_self_^cross^ is similar to *k*_self_^anti^, so that the originally derived *k*_self_^anti^ without considering *k*_self_^cross^ should be reduced by a factor of 5 to yield *k*_self_^cross^ = 2.0 × 10^–10^ cm^3^ molecule^–1^ s^–1^ because [*syn*-CH_3_CHOO]_0_ is about 4 times [*anti*-CH_3_CHOO]_0_. Considering possible errors associated with the branching between *syn*-CH_3_CHOO and *anti*-CH_3_CHOO, we used *k*_self_^cross^ = 2.5 × 10^–10^ cm^3^ molecule^–1^ s^–1^ as an upper limit of *k*_self_^cross^ in the model (Model C). The average values of *k*_self_ derived by Model C is *k*_self_^syn^ = (1.36 ± 0.13) × 10^–10^ cm^3^ molecule^–1^ s^–1^ and *k*_self_^anti^ = (5.2 ± 1.0) × 10^–10^ cm^3^ molecule^–1^ s^–1^. When we used *k*_self_^cross^ ≥ 2.75 × 10^–10^ cm^3^ molecule^–1^ s^–1^ in the model fit, we could not obtain a satisfactory fit. Therefore, a reasonable value of *k*_self_^cross^ is estimated to be (1.5–2.7) × 10^–10^ cm^3^ molecule^–1^ s^–1^, that is *k*_self_^cross^ = (2.1 ± 0.6) × 10^–10^ cm^3^ molecule^–1^ s^–1^. We set *k*_self_^cross^ = 2.1 × 10^–10^ cm^3^ molecule^–1^ s^–1^ (Model D) and refit *k*_self_^syn^ and *k*_self_^anti^. The derived *k*_self_^syn^ and *k*_self_^anti^ are also listed in Table S4 and shown as solid symbols in Figure 3. The average values are *k*_self_^syn^ = (1.38 ± 0.13) × 10^–10^ cm^3^ molecule^–1^ s^–1^ and *k*_self_^anti^ = (6.2 ± 1.0) × 10^–10^ cm^3^ molecule^–1^ s^–1^; these are likely the best fits for the self-reactions.

After considering possible errors, discussed in Section SB, Supporting Information, and assuming *k*_self_^cross^ = (2.1 ± 0.6) × 10^–10^ cm^3^ molecule^–1^ s^–1^ we report rate coefficients *k*_self_^syn^ and *k*_self_^anti^ for the self-reactions of *syn*-CH_3_CHOO and *anti*-CH_3_CHOO to be *k*_self_^syn^ = (1.4 ± 0.3) × 10^–10^ and *k*_self_^anti^ = (6 ± 2) × 10^–10^ cm^3^ molecule^–1^ s^–1^, respectively. The value of *k*_self_^syn^ is similar to the value (1.6 ± _0.6_^0.5^) × 10^–10^ cm^3^ molecule^–1^ s^–1^ reported previously using QCL,^24^ with error limits overlapped. The value of *k*_self_^anti^ is new and is about 4 times that of *k*_self_^syn^.

If we consider the long-range dipole–dipole interaction, the capture rate coefficient derived by Georgievskii and Klippenstein^45^ is
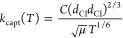
10in which *C* is a constant, *d*_CI_ is the dipole moment of CH_3_CHOO, and μ is the reduced mass. Using this equation and the dipole moment calculated with the B3LYP/cc-pVTZ method (*d*_CI_^syn^ = 4.69 D and *d*_CI_^anti^ = 5.53 D), we obtained *k*_capt_^anti^/*k*_capt_^syn^ = 1.3, much smaller than our observation. Similarly, if the hard-sphere collision is considered with the effective radii r_CI_^syn^ = 2.8 Å and r_CI_^anti^ = 3.4 Å calculated with the “volume” keyword in the Gaussian 16 program, *k*_self_^anti^/*k*_self_^syn^ = 1.5 was derived. The much greater *k*_self_^anti^/*k*_self_^syn^ might be related to the interaction of the terminal O atoms with the two hydrogen atoms in the methyl group of *syn*-CH_3_CHOO, and deserves further theoretical investigations.

### 3.3 Rate Coefficients for the Formation Reaction CH_3_CHI + O_2_

**Figure 4:**
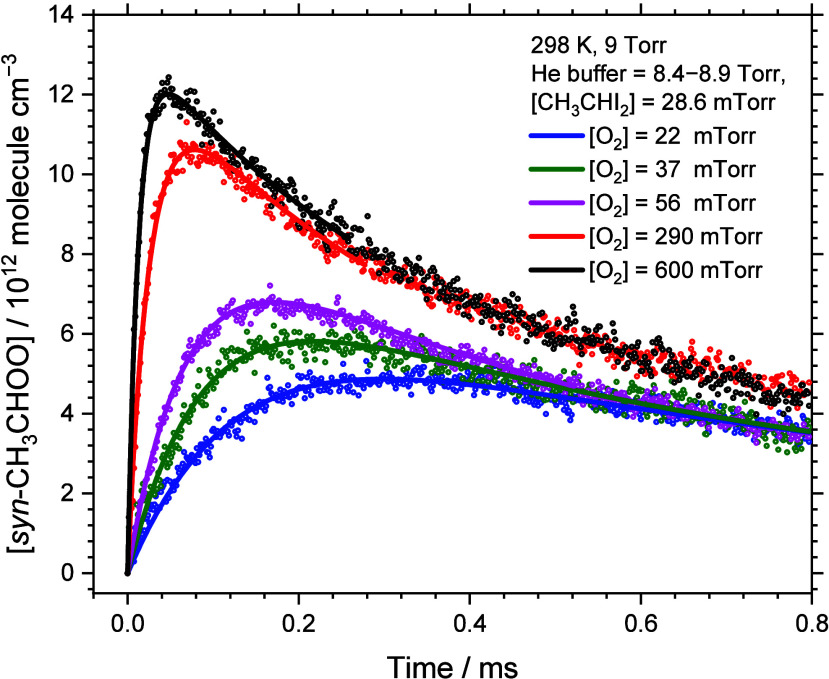
Representative temporal profiles of *syn-*CH_3_CHOO at varied concentrations of O_2_ at 298 K. Signal was integrated over 883.105–883.135 and 883.148–883.185 cm^–1^. [CH_3_CHI_2_] = 28.6 mTorr, [O_2_] = (0.02–0.60) Torr, total pressure *P*_T_ = 9.0 Torr; buffer gas: helium.

During the fitting of the self-reactions, we found that, when we used the literature values *k* = (8.0–8.6) × 10^–12^ cm^3^ molecule^–1^ s^–1^ for the formation of CH_3_CHOO from CH_3_CHI + O_2_ in the fitting,^22,29^ the rising part of the temporal profile could not be fitted well. We hence reinvestigated the formation reaction by using O_2_ with concentrations smaller than those used for self-reactions, so that the rising part could be characterized better. We integrated the absorbance of the band of *syn*-CH_3_CHOO in regions 883.105–883.135 and 883.148–883.185 cm^–1^ to obtain the temporal profile of *syn*-CH_3_CHOO; representative ones are shown in Figure 4.

We fitted the rate coefficient for the formation of CH_3_CHOO from CH_3_CHI + O_2_ according to the model listed in Table S5 by using the branching ratio, the rate coefficients of the cross-reaction between *syn*-CH_3_CHOO and *anti*-CH_3_CHOO, and that of the self-reactions of *syn*-CH_3_CHOO and *anti*-CH_3_CHOO determined in this work. We made the following assumptions in the fitting. (1) The rate coefficients *k*_1_–*k*_6_ are unknown, so we used the same values as those corresponding to CH_2_OO. (2) the branching between CH_3_CHOO:CH_3_CHIOO from the reaction of CH_3_CHI + O_2_ (*k*_form_) was set to be 0.86:0.14 according to Howes et al.^29^ (3) The branching ratio of *syn*-CH_3_CHOO:*anti*-CH_3_CHOO from the reaction of CH_3_CHI + O_2_ was set to 0.80:0.20. (4) The first-order rate coefficient *k*^I^ for the reaction CH_3_CHI + O_2_ was fitted.

**Figure 5:**
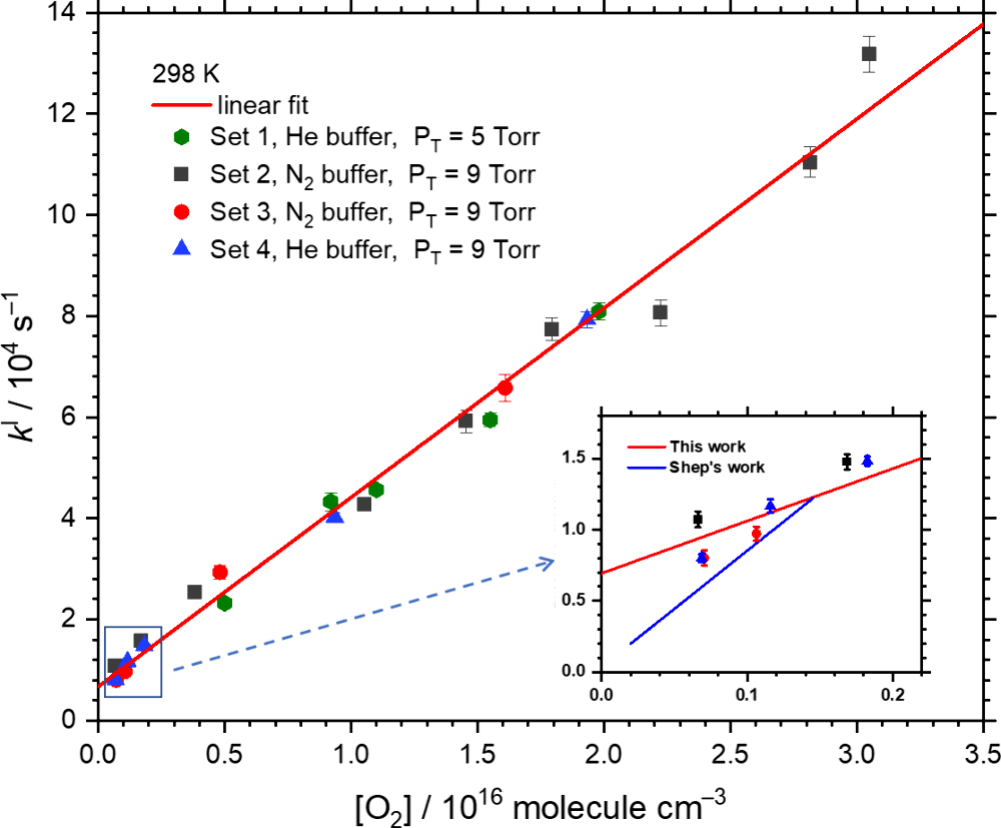
First-order rate coefficient *k*^I^ as a function of [O_2_]. Rate coefficient *k*^I^ was derived by kinetic model fitting. Total pressure 5–9 Torr, *T* = 298 K, [CH_3_CHI_2_]_0_ = (5.6–14.2) × 10^14^ molecules cm^–3^, [O_2_] = (0.07–3.05) × 10^16^ molecules cm^–3^. Linear fit yield the red line with a slope of *k*_form_ = (3.75 ± 0.09) × 10^–12^ cm^3^ molecule^–1^ s^–1^ and an intercept 6690 ± 1300 s^–1^. Slope in the work of Sheps et al.^22^ is shown in the inset as a blue line covering the range of [O_2_] employed in their work.

For this formation reaction, our kinetic analysis was based on mostly the temporal profiles of the IR absorption that monitored only *syn*-CH_3_CHOO; the concentration of *syn*-CH_3_CHOO could be determined as discussed previously. A summary of experimental conditions and fitted first-order rate coefficient *k*^I^ of 23 experiments with [CH_3_CHI]_0_ = (1.3–9.8) × 10^13^ molecules cm^–3^, [O_2_] = (0.7–30.5) × 10^15^ molecules cm^–3^, and total pressure *P*_T_ = 5.0 and 9.0 Torr are listed in Table S6. Representative temporal profiles of *syn*-CH_3_CHOO in experimental set 4 with [CH_3_CHI]_0_ = (2.0–2.4) × 10^13^ molecules cm^–3^ and [O_2_] = (0.7–19.3) × 10^15^ molecules cm^–3^ (0.02–0.60 Torr) at 298 K are depicted as symbols in Figure 4; the fitted temporal evolution are presented with solid lines. The fitted *k*^I^ values as a function of [O_2_] is shown in Figure 5. The bimolecular rate coefficient, *k*_form_, obtained from a linear least-squares fitting of Figure 5, is (3.75 ± 0.09) × 10^–12^ cm^3^ molecule^–1^ s^–1^; the error limit represents one standard deviation in fitting.

For cross-checking, even though the quality and reliability are not as good as those from *syn*-CH_3_CHOO, we also analyzed several temporal profiles of *anti*-CH_3_CHOO, obtained on subtracting the scaled IR profile from the UV profile, as discussed previously. The results of fitted first-order rate coefficients *k*^I^ in set 4 are also listed in Table S6; the averaged deviations from those fitted with temporal profiles of *syn*-CH_3_CHOO are (16 ± 11)% and *k*_form_ = (4.4 ± 0.2) × 10^–12^ cm^3^ molecule^–1^ s^–1^ from these data, consistent with the results derived from the temporal profiles of *syn*-CH_3_CHOO.

Considering the error in estimates of concentrations of *syn*-/*anti*-CH_3_CHOO (20%) that transforms into an error of 11% in *k*_form_, the error in the self-reaction rate coefficient of *syn*-CH_3_CHOO (19%) that transforms into an error of 12% in *k*_form_, the error in the cross-reaction rate coefficient between *syn*-CH_3_CHOO and *anti*-CH_3_CHOO (29%) that transforms into an error of 8% in *k*_form_, and the fitting error of 3%, we estimated the overall uncertainty to be ±18%. Hence the rate coefficient *k*_form_ for the rate coefficient of CH_3_CHI + O_2_ is reported to be (3.8 ± 0.7) × 10^–12^ cm^3^ molecule^–1^ s^–1^.

In Figure 4, results of Sheps et al.^22^ are also indicated as a blue line in the inset for comparison; the range of [O_2_] employed were ∼(0.02–0.14) × 10^16^ molecules cm^–3^. Howes et al.^29^ reported a similar rate coefficient with the range of [O_2_] in their experiments ∼(0.01–0.064) × 10^16^ molecules cm^–3^. Our work covers a much wider range of [O_2_], (0.07–3.05) × 10^16^ molecules cm^–3^. The rate coefficient obtained in this work is approximately 48% the values of (8.0 ± 0.8) × 10^–12^ cm^3^ molecule^–1^ s^–1^ reported by Sheps et al., who employed time-resolved broadband cavity-enhanced UV absorption of CH_3_CHOO for measurement; it is ∼44% the value of (8.6 ± 2.2) × 10^–12^ cm^3^ molecule^–1^ s^–1^ by Howes et al., who employed mass spectroscopy to probe iodine atom. It is unclear why such a large discrepancy exists, but because our work covers a much wider range of [O_2_] and also provides accurate measurements of [*syn*-CH_3_CHOO] and the rate coefficient of self-reactions and the cross-reaction, we expect that our results to be more reliable. Our reported rate coefficient is only about 2.5 times the corresponding rate coefficient for the formation of CH_2_OO from CH_2_I + O_2_, ∼1.5 × 10^–12^ cm^3^ molecule^–1^ s^–1^;^46−50^ this ratio appears to be more reasonable if we consider the size difference of CH_3_CHI (2.6 Å) versus CH_2_I (2.2 Å) and the larger dipole moment of *d*_CI_^CH3CHI^ = 1.39 D versus *d*_CI_^CH2I^ = 0.67 D.

## 4 Conclusions

By utilizing an IR/UV dual-probe system with a quantum-cascade laser near 11 μm to probe *syn*-CH_3_CHOO and a 335 nm solid-state laser to probe *syn*-/*anti*-CH_3_CHOO, and an additional light-emitting diode at 286 nm to quantify the concentration variation of the precursor CH_3_CHI_2_, we were able to obtain concentration profiles of *syn*-CH_3_CHOO and *anti*-CH_3_CHOO and characterize the branching between *syn*-CH_3_CHOO and *anti*-CH_3_CHOO to be (80 ± 10): (20 ± 10) from CH_3_CHI + O_2_, larger than the previous result of ∼70:30 reported by Sheps et al.,^22^ but smaller than the ratio 90:10 reported by Taatjes et al.;^10^ these literature values are within our error limit. By model-fitting the temporal profiles of *syn*-CH_3_CHOO and *anti*-CH_3_CHOO, we estimated the rate coefficient of the cross-reaction between *syn*-CH_3_CHOO and *anti*-CH_3_CHOO to be *k*_self_^cross^ = (2.1 ± 0.6) × 10^–10^ cm^3^ molecule^–1^ s^–1^ and determined the rate coefficient for the self-reaction of *syn*-CH_3_CHOO to be *k*_self_^syn^ = (1.4 ± 0.3) × 10^–10^ cm^3^ molecule^–1^ s^–1^, consistent with our previous report of (1.6 ± _0.6_^0.5^) × 10^–10^ cm^3^ molecule^–1^ s^–1^.^24^ The determination of the rate coefficient for the self-reaction of *anti*-CH_3_CHOO, *k*_self_^anti^ = (6 ± 2) × 10^–10^ cm^3^ molecule^–1^ s^–1^, is new; it is more than 4 times of *k*_self_^syn^, likely because the dipole moment of *anti*-CH_3_CHOO is greater than *syn*-CH_3_CHOO and the OO moiety of *anti*-CH_3_CHOO is free as compared with that of *syn*-CH_3_CHOO. With the branching and the rate coefficients of cross-reaction and self-reactions determined, we investigated the formation rate coefficient of CH_3_CHOO from CH_3_CHI + O_2_ and determined *k*_form_ = (3.8 ± 0.7) × 10^–12^ cm^3^ molecule^–1^ s^–1^ at 5–9 Torr and 298 K, which is approximately 45% of the two previously reported values. It is unclear why such a discrepancy exists, but because our work covers a much wider range of [O_2_] and also provides accurate measurements of [*syn*-CH_3_CHOO] and the cross-reaction and self-reaction rates of CH_3_CHOO, we expect our results to be more reliable. A smaller rate coefficient for the formation of CH_3_CHOO might affect the kinetic analysis of reactions of CH_3_CHOO with other atmospheric species.

This work demonstrates the advantages of combining both UV and high-resolution IR absorption for kinetic studies of conformation-specific Criegee intermediates. In the case of CH_3_CHOO, the IR absorption provides direct measurements of *syn*-CH_3_CHOO and the UV absorption provides temporal profiles of *syn*-/*anti*-CH_3_CHOO, from which temporal profiles with accurate concentration measurements of *anti*-CH_3_CHOO could be derived, which are critical for precise measurements of rate coefficient. This method is suitable for direct investigations on reactions of *syn*-CH_3_CHOO and *anti*-CH_3_CHOO with atmospheric species.

## Supporting Information Available

Concentration measurements of [CH_3_CHI]_0_; preliminary kinetic analysis and error analysis of the self-reactions of *syn*- and *anti*-CH_3_CHOO; experimental conditions and measured parameters to derive the branching ratio *a*; experimental conditions and derived rate coefficients for the self-reactions of *syn*-CH_3_CHOO (*k*_self_^syn^) and *anti*-CH_3_CHOO (*k*_self_^anti^) with various models; kinetic model for fitting the rate coefficients of self-reactions of *syn*-/*anti*- CH_3_CHOO; kinetic model for fitting the rate coefficient of the reaction CH_3_CHI + O_2_; experimental conditions and the fitted first-order rate coefficient (*k*^I^) of CH_3_CHI + O_2_ using various models; the intensity of the 286 nm light before and after photolysis of CH_3_CHI_2_ at 248 nm; representative plot of [A_335_]^−1^ versus reaction period at 298 K; estimation of *k*_self_^syn^ and *k*_self_^anti^ from the plot of [CH_3_CHOO]^−1^ vs reaction period; and comparison of *k*_self_^syn^ and *k*_self_^anti^ derived from the second-order fit and the model fit (PDF)
