# Supplementary material for: Rate Coefficient and Branching Ratio for the Formation of Criegee Intermediate Syn-/Anti-CH3CHOO from CH3CHI + O2 and the Self-Reaction of Syn-/Anti-CH3CHOO Determined with Simultaneous IR/UV Probes
Source: J Phys Chem A. 2024 Oct 20;128(43):9453–61. doi: 10.1021/acs.jpca.4c06588 (PMC11533191; doi:10.1021/acs.jpca.4c06588)
Supplement: Supplementary file 1 — jp4c06588_si_001.pdf [file jp4c06588_si_001.pdf]

Supporting Information

Rate Coefficient and Branching Ratio for the Formation of Criegee Intermediate *syn*-/*anti*-CH_3CHOO from $CH_3CHI + O_2$ and the Self-Reaction of *syn*-/*anti*-CH_3CHOO Determined with Simultaneous IR/UV Probes

Tang-Yu Kao,[†] Chen-An Chung,[†] and Yuan-Pern Lee[†,‡*]

[†]*Department of Applied Chemistry and Institute of Molecular Science, National Yang Ming Chiao Tung University, His nchu 300093, Taiwan*
[‡]*Center for Emergent Functional Matter Science, National Yang Ming Chiao Tung University, Hsinchu 300093, Taiwan*

**E-mail: yplee@nycu.edu.tw* (Y.-P. Lee)

Table of Contents

Section SA. Concentration measurements of $[CH_3CHI]_0$ S2

Section SB. Preliminary kinetic analysis and error analysis of the self-reactions of *syn*- and *anti*-CH_3CHOO S3

Table S1. Experimental conditions and measured parameters to derive the branching ratio ... S6

Table S2. Experimental conditions and estimated rate coefficients for the self-reactions of *syn*-CH_3CHOO (k_{self}^{syn}) and *anti*-CH_3CHOO (k_{self}^{anti}) without considering the cross-reaction S7

Table S3. Kinetic model for fitting the rate coefficient of the reaction $CH_3CHI + O_2$ S8

Table S4. Experimental conditions and derived rate coefficients for the self-reactions of *syn*-CH_3CHOO (k_{self}^{syn}) and *anti*-CH_3CHOO (k_{self}^{anti}) after considering the cross-reaction..... S9

Table S5. Kinetic model for fitting the rate coefficients of self-reactions of *syn*-/*anti*-CH_3CHOO S10

Table S5. Experimental conditions and the fitted first-order rate coefficient (k^I) of $CH_3CHI + O_2$ S11

Figure S1. The intensity of the 286-nm light before and after photolysis of CH_3CHI_2 at 248 nm S12

Figure S2. Representative plot of $[A_{335}]^{-1}$ versus reaction period at 298 K........ S13

Figure S3. Estimation of k_{self}^{syn} and k_{self}^{anti} from the plot of $[CH_3CHOO]^{-1}$ vs. reaction period........ S14

Figure S4. Comparison of k_{self}^{syn} and k_{self}^{anti} derived from the second-order fit and the fitt in Model A........ S15

References S16

SA. Concentration measurements of $[CH_3CHI]_0$

To quantify the concentration of the precursor CH_3CHI_2, we probed $[CH_3CHI_2]$ with light near 286 nm from a LED. Typical traces before and after photolysis are presented in Figure S1. The light intensity with no CH_3CHI_2, I_0, is indicated in Figure S1; after introducing CH_3CHI_2, the intensity drops to I_1, which corresponds to $[CH_3CHI_2]_0$. Upon photolysis, the DC signal increases to I_2; the increase is due to the decrease in $[CH_3CHI_2]$. We performed two types of experiments to confirm the variations in signal. As shown in Figure S1, the red trace was obtained when no O_2 was added and the black trace was obtained when O_2 was added to the system with CH_3CHI_2. The red trace showed a slow decay, which might be due to the recombination of CH_3CHI with I to reproduce CH_3CHI_2. The black curve has an additional negative component that decayed within 0.5 ms; this component is ascribed to the absorption of the Criegee intermediate CH_3CHOO, which decays mainly due to its self-reaction. Extrapolation from the later part of the curve with O_2 (blue line) to time zero yields I_2, which is slightly larger than the value obtained from experiments without O_2 (green line). The difference might be due to the absorption of CH_3CHI when no O_2 was present, but we are unable to confirm this; this deviation is considered as the uncertainty in the estimate of $\Delta[CH_3CHI_2]$. Typically, the error is within 6 %.

The difference in absorbance ΔA upon photolysis corresponds to the loss of $[CH_3CHI_2]$ due to photolysis.

$$\Delta A = -\ln (I_1/I_2) = \sigma_{CH3CHI2} \times l \times \Delta[CH_3CHI_2], \qquad \text{(S1)}$$

in which I_1 is the light intensity before photolysis of the precursor, and I_2 is the light intensity after photolysis, $\sigma_{CH3CHI2}$ is the absorption cross section of CH_3CHI_2 in cm^2 $molecule^{-1}$, which is 3.56×10^{-18} cm^2 $molecule^{-1}$ at 286 nm,[1] l is the absorption path length (87.0 cm), and $\Delta[CH_3CHI_2]$ is the decrease in concentration (in molecule cm^{-3}) of the precursor CH_3CHI_2 after photolysis. Assuming a photolysis yield of 1, we derived $[CH_3CHI]_0$ to be $\Delta[CH_3CHI_2]$, as listed in Table S1 along with experimental conditions.

SB. Preliminary kinetic analysis and error analysis of the self-reactions of *syn*- and *anti*-CH_3CHOO

According to Section 3.1 in the main text, [*syn*-CH_3CHOO]$_0$ could be determined from either Method A (using Δ[CH_3CHI_2], UV profile, the reported σ_{syn} and σ_{anti} at 335 nm, and $a + b = 0.86$) or Method B (using UV and IR profiles and the reported σ_{syn} and σ_{anti} at 335 nm). Because the reaction of $CH_3CHI + O_2$ typically takes 3~7 μs to reach 90% completion, the [CH_3CHOO]$_0$, hence A_{335}^0, the absorbance at 335 nm, could not be determined directly upon irradiation at 248 nm. We assumed that the key loss of CH_3CHOO is due to its self-reaction, so we plotted $1/A_{335}$ versus time and extrapolated the value to $t = 0$ to obtain A_{335}^0, as shown in Figure S2.

Some representative plots of [*syn*-CH_3CHOO]$^{-1}$ vs. t (time) are shown in Figure S3a; the initial part (t = 0−15 μs) was not used in the fitting because of the incomplete formation of CH_3CHOO. Assuming that only the self-reaction (no cross-reaction) is the major loss of *syn*-CH_3CHOO,

syn-CH_3CHOO + *syn*-CH_3CHOO → products (k_{self}^{syn}) (S2)

the slopes in Figure S3a yield an estimate of $2k_{self}^{syn}$, twice the rate coefficient for the self-reaction of *syn*-CH_3CHOO. A summary of experimental conditions and the results are shown in Table S2; a statistical distribution of these measurements of k_{self}^{syn} (black squares) appears in Figure S4a. The average of 9 experiments with [*syn*-CH_3CHOO]$_0$ = (2.1−10.2)×10^{13} molecule cm^{-3}, P = 5.0−30.0 Torr, and T = 298 K yields an estimate of k_{self}^{syn} = (1.8 ± 0.3)×10^{-10} cm^3 $molecule^{-1}$ s^{-1}; the uncertainty limits represent one standard deviation in fitting.

As discussed previously, by subtracting the scaled IR signal from the UV signal, we derived the temporal profile of *anti*-CH_3CHOO, as shown in Figure 2c. We plotted similarly [*anti*-CH_3CHOO]$^{-1}$ vs. t (time) in Figure S3b; the initial part (t = 0−10 μs) was not used because of the incomplete formation of CH_3CHOO. Assuming that only the self-reaction (no cross-reaction) is the major loss of *anti*-CH_3CHOO,

anti-CH_3CHOO + *anti*-CH_3CHOO → products (k_{self}^{anti}) (S3)

the slopes in Figure S3b yield an estimate of $2k_{self}^{anti}$, twice the rate coefficient for the self-reaction of *anti*-CH_3CHOO. A summary of the fitted results are shown in Table S2; a statistical distribution of these measurements of k_{self}^{anti} (black squares) appears in Figure S4b. The average of 9 experiments with [*anti*-CH_3CHOO]$_0$ = $(0.8-3.8)\times10^{13}$ molecule cm^{-3} yields an estimate of $k_{self}^{anti} = (10.3 \pm 1.4)\times10^{-10}$ cm^3 $molecule^{-1}$ s^{-1}; the uncertainty limits represent one standard deviation in fitting.

For more accurate determination, we performed a model fit. This model, Model A, included the three channels of the formation reaction $CH_3CHI + O_2$, the self-reaction of *syn*- and *anti*-CH_3CHOO (but no cross-reaction), reactions of *syn/anti*-CH_3CHOO + I and other minor reactions involving reactions with I atoms, as listed in Table S3. In this model, we did not specify the products of the reaction of $CH_3CHIOO + O_2$, which might produce $CH_3CHI + O_2$, CH_3CHIOO, and CH_3CHIO + IO, as they have insignificant effects on k_{self}^{syn} and k_{self}^{anti}, which are much larger than k_1 and k_2. The pressure dependence of this reaction was also not considered under our experimental conditions. The reaction CH_3CHOO + IO was not included because the effect of this reaction on k_{self}^{syn} and k_{self}^{anti} is expected to be small, as $[IO]_0$ is smaller than $[I]_0$.

In fitting k_{self}^{syn} and k_{self}^{anti} , some assumptions were made: (1) The rate coefficients k_1–k_5 in Table S3 are unknown, so we used the same values as those corresponding to CH_2OO. (2) The formation rate coefficient of $CH_3CHI + O_2$ (k_{form}) was set to be 3.8×10^{-12} cm^3 $molecule^{-1}$ s^{-1} (to be dicussed later). (3) the branching ratio between CH_3CHOO : CH_3CHIOO from the reaction of $CH_3CHI + O_2$ (k_{form}) was set to be 0.86 : 0.14 according to Howes et al.[2] (4) The branching ratio of *syn*-CH_3CHOO : *anti*-CH_3CHOO from the reaction of $CH_3CHI + O_2$ was set at 0.80 : 0.20. (5) We allowed ±18% variation of the rate coefficients of the three channels in the formation reaction, k_{form}^{a}, k_{form}^{b} and k_{form}^{c}, in fitting. The derived k_{self}^{syn} and k_{self}^{anti} from the model fit are compared with results from the second-ordert fit in Table S2 and plotted in Figure S4 for comparison. The average values of the model fit gave $k_{self}^{syn} = (1.5 \pm 0.2)\times10^{-10}$ cm^3 $molecule^{-1}$ s^{-1} and $k_{self}^{anti} = (10.2 \pm 1.5)\times10^{-10}$ cm^3 $molecule^{-1}$ s^{-1}. The fitted rate coefficients decreased by

~17% for k_{self}^{syn} and 1% for k_{self}^{anti} as compared to those derived from the second-ordert fit. The value of k_{self}^{syn} is significantly smaller than that estimated from the second-order plot because secondary reactions were considered. The value of k_{self}^{anti} is the same as that estimated from the second-order plot because the rate coefficient is large so that it is less affected by secondary reactions.

The discussion of the fitting after considering the cross-reaction with various rate coefficients k_{self}^{cross} (Models B−D) is described in the main text. The experimental conditions and fitted results are listed in Table S4. Values of k_{self}^{syn} remain nearly constant in three models because *syn*-CH_3CHOO is the major conformer. Considering errors in estimates of concentrations of *syn-/anti*-CH_3CHOO (±20 %) that transforms into an error of ±12 % in k_{self}^{syn}, the error in the rate coefficient of $CH_3CHI + O_2$ (±18 %) that transforms into an error of ±10 % in k_{self}^{syn}, the error induced by the uncertainty of k_{self}^{cross} (±3%), and the fitting error (±10 %), we estimated the overall uncertainty for k_{self}^{syn} to be ±19 %. Rate coefficients k_{self}^{syn} for the self-reactions of *syn*-CH_3CHOO is hence reported to be $(1.4 \pm 0.3)\times10^{-10}$ cm^3 $molecule^{-1}$ s^{-1}. This value is consistent with the value $(1.6\pm^{0.5}_{0.6})\times10^{-10}$ cm^3 $molecule^{-1}$ s^{-1} reported previously using QCL IR absorption and an estimated [*syn*-CH_3CHOO].[3] For k_{self}^{anti}, the derived values $(5.2–7.4)\times10^{-10}$ cm^3 $molecule^{-1}$ s^{-1} depend on k_{self}^{cross}; the deviation of k_{self}^{anti} is about $\pm$ 1×10^{-10} cm^3 $molecule^{-1}$ s^{-1}, ~16%. Considering errors in estimates of concentrations of *syn-/anti*-CH_3CHOO (±20 %) that transforms into an error of ±16 % in k_{self}^{anti}, the error in the rate coefficient of $CH_3CHI + O_2$ (±18 %) that transforms into an error of ±11 % in k_{self}^{anti}, the error induced by the uncertainty of k_{self}^{cross} (±16%), and the fitting error (±15 %), we estimated the overall uncertainty for k_{self}^{anti} to be ±28 %. Rate coefficients k_{self}^{anti} for the self-reactions of *syn*-CH_3CHOO is hence first reported to be $(6 \pm 2)\times10^{-10}$ cm^3 $molecule^{-1}$ s^{-1}.

Table S1. Experimental Conditions and Measured Parameters to Derive the Branching Ratio

expt.	P_T /Torr	$[CH_3CHI_2]$ /10^{15} [a]	yield /%	$[CH_3CHI]_0$ /10^{13} [a]	$[O_2]$ /10^{17} [a]	$1/A_{335}^0$	Method A[b] *a*	Method A[b] *a*/(*a*+*b*)	Method B[c] *a*/(*a*+*b*)
1	5.0	1.3	7.2	9.4	1.1	6.5	0.76	0.89	0.80
2	5.0	1.3	6.5	8.5	1.4	7.7	0.54	0.64	0.58
3	5.0	1.3	6.8	8.8	0.9	7.1	0.66	0.77	0.79
4	5.0	1.3	7.3	9.5	1.2	6.5	0.72	0.83	0.80
5	5.0	1.3	6.9	9.0	1.5	6.9	0.71	0.82	0.70
6	5.0	0.5	6.6	3.3	1.2	18.8	0.70	0.81	0.80
7	5.0	0.7	6.8	4.8	1.2	13.1	0.68	0.79	0.75
8	9.0	2.2	6.1	13.4	2.1	4.7	0.70	0.81	0.80
9	9.0	1.5	6.5	9.8	1.7	6.2	0.77	0.89	0.83
10	9.0	1.7	6.5	11.0	1.7	5.6	0.66	0.77	0.60
						Ave.[d]	***0.69***	***0.80***	***0.74***
						Dev.[e]	± ***0.06***	± ***0.07***	± ***0.09***

[a] In molecule cm^{-3}. [b]Using Equation S7 according to measured $[CH_3CHI]_0$ and A_{335}^0. [c]Comparison of deconvoluted profiles of *syn*- and *anti*-CH_3CHOO from UV and IR profiles; see text. [d]Average value. [e]Standard deviation in averaging.

Table S2. Experimental Conditions and Estimated Rate Coefficients for the Self-reactions of *syn*-CH_3CHOO (k_{self}^{syn}) and *anti*-CH_3CHOO (k_{self}^{anti}) without Considering the Cross-reaction

expt.	P_{total} /Torr	[CH_3CHI_2] /$10^{15\ c}$	[CH_3CHI]$_0$ /$10^{13\ c}$	[O_2]$_0$ /$10^{16\ c}$	[He] /$10^{16\ c}$	second-order[a] $k_{self}^{syn\ d}$	second-order[a] $k_{self}^{anti\ d}$	model A[b] $k_{self}^{syn\ d}$	model A[b] $k_{self}^{anti\ d}$
1	5.0	0.5	3.7	12.1	4.2	2.3	11.2	1.7	9.0
2	5.0	0.8	5.1	12.2	4.3	2.1	10.8	1.5	10.2
3	5.0	1.2	7.3	12.4	4.3	1.9	9.8	1.4	8.9
4	5.0	1.5	8.8	11.9	4.6	1.5	8.5	1.3	7.9
5	9.0	2.2	13.4	21.6	7.6	1.4	8.9	1.3	13.1
6	9.0	1.5	9.8	17.3	12.2	1.6	10.1	1.5	10.5
7	9.0	1.7	11.0	17.5	12.2	1.5	9.8	1.5	10.4
8	9.0	2.5	15.8	20.1	9.2	2.2	13.0	1.7	11.0
9	30.0	1.7	10.9	42.6	55.0	1.7	11.0	1.4	10.8
					Ave.[e]	***1.8***	***10.3***	***1.5***	***10.2***
					Dev.[f]	**± *0.3***	**± *1.4***	**± *0.2***	**± *1.5***

[a]Derived from the slope of a plot of [CH_3CHOO]$^{-1}$ vs. *t*. [b]Using a model listed in Table S3; see text. [c]In molecule cm^{-3}. [d]In unit of 10^{-10} cm^3 $molecule^{-1}$ s^{-1}. [e]Average value. [f]Standard deviation in averaging.

Table S3. Kinetic Model for Fitting the Rate Coefficients of Self-reactions of *Syn-/Anti-*CH_3CHOO

reaction		rate coefficient[a,b]	reference
$CH_3CHI + O_2 \rightarrow$ *syn*-CH_3CHOO + I	k_{form}^{a}	$x \times y \times 3.7{\times}10^{-12}$ (± 18 %)<br>$x = 0.86$; $y = 0.80$	[4], [2]
$CH_3CHI + O_2 \rightarrow$ *anti*-CH_3CHOO + I	k_{form}^{b}	$x \times (1 - y) \times 3.7{\times}10^{-12}$ (± 18 %)	[**4**], [2]
$CH_3CHI + O_2 \rightarrow CH_3CHIOO$	k_{form}^{c}	$(1 - x) \times 3.7{\times}10^{-12}$ (± 18 %)	[2]
2 *syn*-$CH_3CHOO \rightarrow$<br>2 $CH_3CHO + O_2$	k_{self}^{syn}	fitted	
2 *anti*-$CH_3CHOO \rightarrow$<br>2 $CH_3CHO + O_2$	k_{self}^{anti}	fitted	
syn-CH_3CHOO + I → products	k_1	9.0×10^{-12}	[5]
anti-CH_3CHOO + I → products	k_2	9.0×10^{-12}	[5]
$CH_3CHIOO + I \rightarrow CH_3CHIO + IO$	k_3	3.5×10^{-11}	[5]
2 $CH_3CHIOO \rightarrow$ 2 $ICH_3CHO + O_2$	k_4	9.0×10^{-11}	[5]
$CH_3CHIO \rightarrow CH_3CHO + I$	k_5	10^{6} s^{-1}	[6]
2 IO → $I_2 + O_2$	k_6	9.9×10^{-11}	[5]

[a]Listed second-order rate coefficients are in cm^3 $molecule^{-1}$ s^{-1}. [b]x is the branching ratio of *syn-/anti*-CH_3CHOO in total products and y is the branching ratio of *syn*-CH_3CHOO in total CH_3CHOO.

Table S4. Experimental Conditions and Derived Rate Coefficients for the Self-reactions of *syn*-CH_3CHOO (k_{self}^{syn}) and *anti*-CH_3CHOO (k_{self}^{anti}) after considering the cross-reaction

expt.	P_{total} /Torr	$[CH_3CHI]_0$ /$10^{13\ d}$	$[O_2]_0$ /$10^{16\ d}$	[He] /$10^{16\ d}$	model B[a]		model C[b]		model D[c]	
					$k_{self}^{syn\ e}$	$k_{self}^{anti\ e}$	$k_{self}^{syn\ e}$	$k_{self}^{anti\ e}$	$k_{self}^{syn\ e}$	$k_{self}^{anti\ e}$
1	5.0	3.7	12.1	4.2	1.6	6.8	1.6	4.6	1.6	5.5
2	5.0	5.1	12.2	4.3	1.5	7.5	1.4	5.5	1.5	6.2
3	5.0	7.3	12.4	4.3	1.4	6.7	1.3	4.9	1.3	5.6
4	5.0	8.8	11.9	4.6	1.3	5.5	1.2	3.1	1.3	4.6
5	9.0	13.4	21.6	7.6	1.2	9.3	1.2	6.4	1.2	8.0
6	9.0	9.8	17.3	12.2	1.4	7.1	1.3	5.3	1.3	6.3
7	9.0	11.0	17.5	12.2	1.5	7.8	1.4	5.8	1.4	6.0
8	9.0	15.8	20.1	9.2	1.5	8.4	1.5	5.8	1.5	6.9
9	30.0	10.9	42.6	55.0	1.4	7.6	1.3	5.4	1.3	6.6
				Ave.[e]	***1.42***	***7.4***	***1.36***	***5.2***	***1.38***	***6.2***
				Dev.[f]	± ***0.12***	± ***1.1***	± ***0.13***	± ***1.0***	± ***0.13***	± ***1.0***

[a]Derived from a model listed in Table S3 with the cross-reaction added ($k_{self}^{cross} = 1.5\times10^{-10}$ cm^3 $molecule^{-1}$ s^{-1}); see text. [b]Derived from a model listed in Table S3 with the cross-reaction added ($k_{self}^{cross} = 2.5\times10^{-10}$ cm^3 $molecule^{-1}$ s^{-1}); see text. [c]Derived from a model listed in Table S3 with the cross-reaction added ($k_{self}^{cross} = 2.1\times10^{-10}$ cm^3 $molecule^{-1}$ s^{-1}); see text. [d]In molecule cm^{-3}. [d]In unit of 10^{-10} cm^3 $molecule^{-1}$ s^{-1}. [e]Average value. [f]Standard deviation in averaging.

Table S5. Kinetic model for fitting the Rate Coefficient of the Reaction $CH_3CHI + O_2$

reaction		rate coefficient[a,b]	reference
$CH_3CHI + O_2 \rightarrow$ *syn*-CH_3CHOO + I	k_{form}^{a}	$x \times y \times k^{I}$ (fitted)<br>$x = 0.86$; $y = 0.80$	[4], [2]
$CH_3CHI + O_2 \rightarrow$ *anti*-CH_3CHOO + I	k_{form}^{b}	$x \times (1-y) \times k^{I}$ (fitted)	[4], [2]
$CH_3CHI + O_2 \rightarrow CH_3CHIOO$	k_{form}^{c}	$(1-x) \times k^{I}$ (fitted)	[2]
2 *syn*-$CH_3CHOO \rightarrow 2\ CH_3CHO + O_2$	k_{self}^{syn}	$(1.4 \pm 0.3)\times10^{-10}$	This work
2 *anti*-$CH_3CHOO \rightarrow 2\ CH_3CHO + O_2$	k_{self}^{anti}	$(6 \pm 2)\times10^{-10}$	This work
anti-CH_3CHOO + *anti*-CH_3CHOO $\rightarrow 2\ CH_3CHO + O_2$	k_{self}^{cross}	$(2.1 \pm 0.6)\times10^{-10}$	This work
syn-CH_3CHOO + I $\rightarrow CH_3CHO$ + IO	k_1	9.0×10^{-12}	[5]
anti-CH_3CHOO + I $\rightarrow CH_3CHO$ + IO	k_2	9.0×10^{-12}	[5]
CH_3CHIOO + I $\rightarrow ICH_3CHO$ + IO	k_3	3.5×10^{-11}	[5]
2 $CH_3CHIOO \rightarrow 2\ ICH_3CHO + O_2$	k_4	9.0×10^{-11}	[5]
$ICH_3CHO \rightarrow CH_3CHO$ + I	k_5	10^{6} s^{-1}	[6]
2 IO $\rightarrow I_2 + O_2$	k_6	9.9×10^{-11}	[5]

[a]Listed second-order rate coefficients are in cm^3 $molecule^{-1}$ s^{-1}. [b]x is the branching ratio of *syn*-/*anti*-CH_3CHOO in total products and y is the branching ratio of *syn*-CH_3CHOO in total CH_3CHOO. k^{I} is the firs-order rate coefficient to be fitted.

Table S6. Experimental Conditions and the Fitted First-order Rate Coefficient (k^{I}) of $CH_3CHI + O_2$

set	expt.	P_T /Torr	$[CH_3CHI_2]$ /$10^{14\,c}$	$[CH_3CHI]_0$ /$10^{13\,c}$	$[N_2]$ /$10^{16\,c}$	[He] /$10^{16\,c}$	$[O_2]$ /$10^{16\,c}$	$k^{I\,a}$ /10^4 s^{-1}	$k^{I\,b}$ /10^4 s^{-1}
1	1	5.0	14.2	9.5	0	15.6	0.50	2.3	
	2	5.0	14.2	9.5	0	15.2	0.92	4.3	
	3	5.0	14.2	9.8	0	14.9	1.10	4.6	
	4	5.0	14.2	9.2	0	14.5	1.55	5.9	
	5	5.0	14.2	9.5	0	14.1	1.98	8.1	
2	6	9.0	9.2	2.2	28.8	0	0.07	1.1	
	7	9.0	9.2	2.2	28.7	0	0.17	1.5	
	8	9.0	9.2	2.3	28.6	0	0.38	2.5	
	9	9.0	9.2	2.2	28.2	0	1.00	4.3	
	10	9.0	9.2	2.4	27.7	0	1.45	6.0	
	11	9.0	9.2	2.3	27.1	0	1.80	7.7	
	12	9.0	9.2	2.2	26.7	0	2.22	8.1	
	13	9.0	9.2	2.0	26.1	0	2.81	11.0	
	14	9.0	9.2	2.0	25.5	0	3.05	13.2	
3	15	9.0	5.6	1.4	28.9	0	0.07	0.8	
	16	9.0	5.6	1.4	28.8	0	0.11	1.0	
	17	9.0	5.6	1.3	28.4	0	0.48	3.0	
	18	9.0	5.6	1.4	27.1	0	1.60	6.6	
4	19	9.0	9.2	2.2	0	28.8	0.07	0.8	1.0
	20	9.0	9.2	2.2	0	28.8	0.12	1.2	1.2
	21	9.0	9.2	2.4	0	28.7	0.18	1.5	1.9
	22	9.0	9.2	2.2	0	28.2	0.93	4.0	4.3
	23	9.0	9.2	2.0	0	27.0	1.93	8.1	9.1

[a]Fitted from temporal profiles of *syn*-CH_3CHOO according to the model listed in Table S4. [b]Fitted from the derived temporal profiles of *anti*-CH_3CHOO. [c] in unit of molecule cm^{-3}.

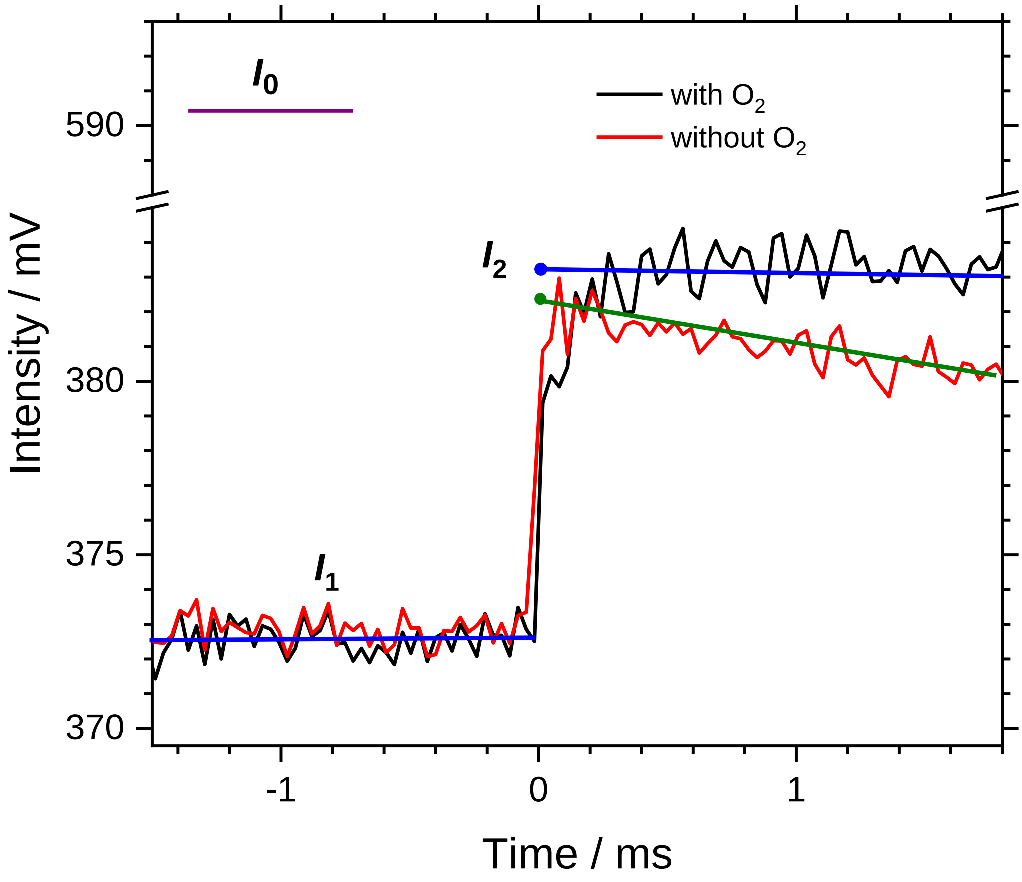

Figure S1. The intensity of the 286-nm light before and after photolysis of CH_3CHI_2 at 248 nm. The light intensity with no CH_3CHI_2 in the system is I_0; that with CH_3CHI_2 is I_1, which is smaller than I_0 because of absorption. Photolysis took place at time zero. The black and red curves are from experiments at the same $[CH_3CHI_2]_0$ with and without O_2 added, respectively; CH_3CHI reacts with O_2 to form CH_3CHOO. The abrupt increase in intensity is due to the loss of CH_3CHI_2. The slow decrease in intensity of the red trace after photolysis is likely due to the recombination reaction $I + CH_3CHI \rightarrow CH_3CHI_2$, and the additional rapid-decay component in the black trace after photolysis is due to the weak absorption of $[CH_3CHOO]$, which decays within 0.5 ms. Extrapolation to time zero (blue and green lines) gives I_2, which corresponds to $[CH_3CHI_2]$ after photolysis. $\Delta[CH_3CHI_2]$ could hence be derived from $ln\ (I_1/I_2)$. For these traces, $[CH_3CHI_2]_0 = 1.5 \times 10^{15}$ molecule cm^{-3} and $\Delta[CH_3CHI_2] = 9.5 \times 10^{13}$ molecule cm^{-3}.

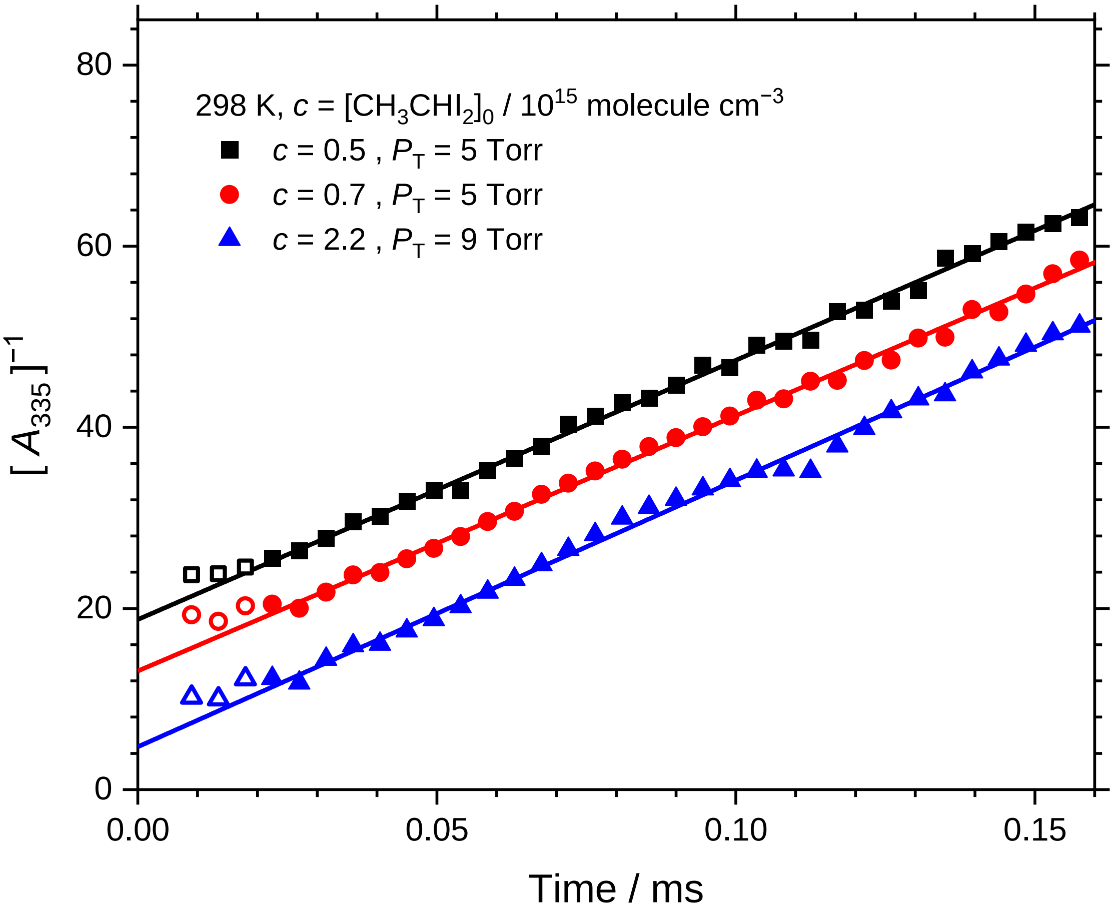

Figure S2. Representative plot of $[A_{335}]^{-1}$ versus reaction period at 298 K. We assumed that the key loss of CH_3CHOO was due to its self-reaction, hence the value of $1/A_{335}$ extrapolated to reaction period Time = 0 gave $1/A_{335}^0$. Only data of solid symbols were used in the linear fit; the open symbols that deviated from the line due to the slow formation of CH_3CHOO were not used.

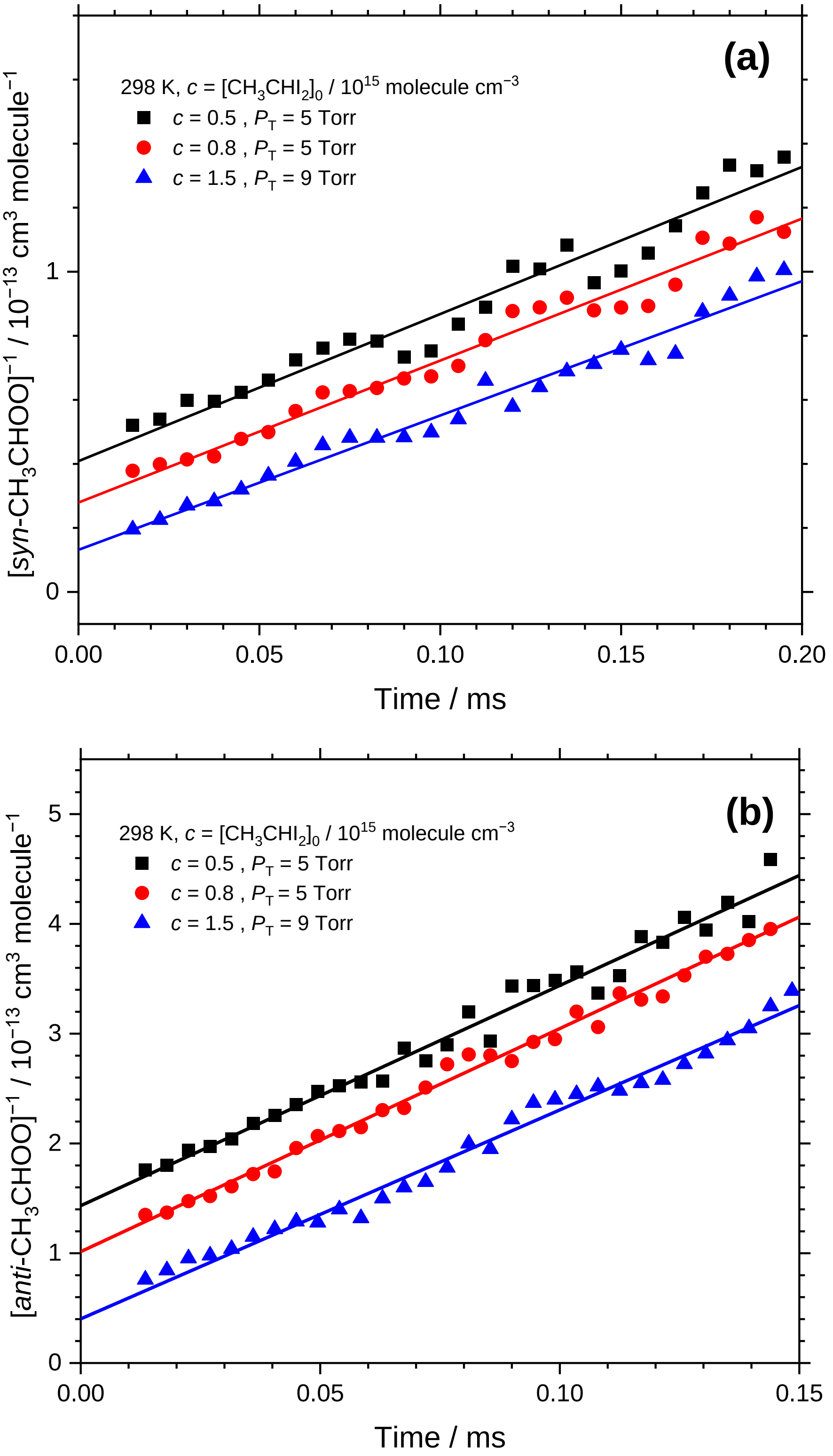

Figure S3. Estimation of k_{self}^{syn} and k_{self}^{anti} from the plot of $[CH_3CHOO]^{-1}$ vs. reaction period. (a) [*syn*-$CH_3CHOO]^{-1}$ vs. reaction period (Time); (b) [*anti*-$CH_3CHOO]^{-1}$ vs. Time. The data in the initial period (not shown) were not used in the fitting because of the slower formation of CH_3CHOO. The slopes in (a) and (b) yield estimates of $2k_{self}^{syn}$ and $2k_{self}^{anti}$, respectively.

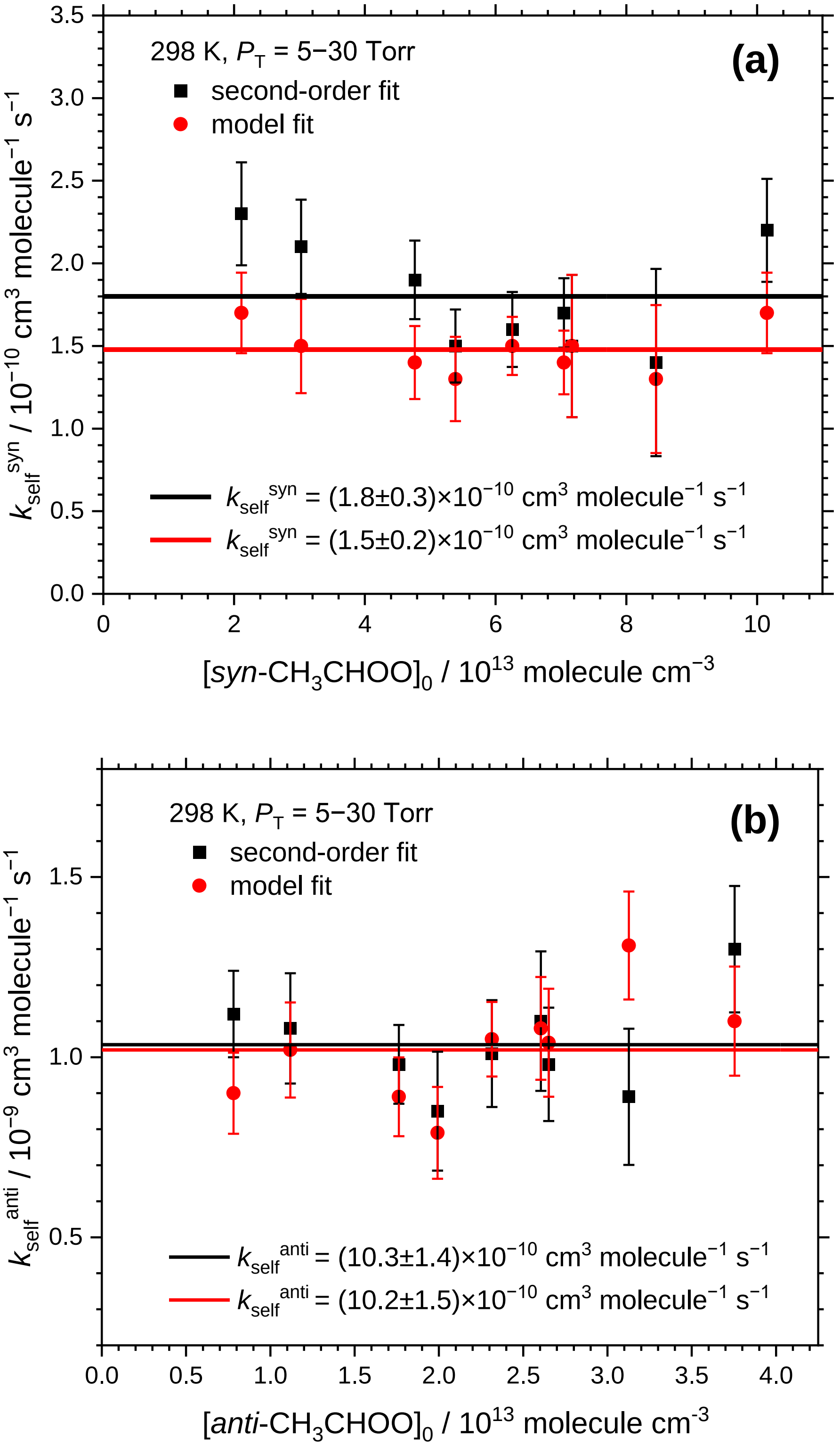

Figure S4. Comparison of k_{self}^{syn} and k_{self}^{anti} derived from the second-order fit and the fit in Model A. (a) k_{self}^{syn}, with [*syn*-CH_3CHOO]$_0$ = (2.1−10.2)×10^{13} molecule cm^{-3}, P_T = 5.0−30.0 Torr, and T = 298 K. (b) k_{self}^{anti}, with [*anti*-CH_3CHOO]$_0$ = (0.8−3.8)×10^{13} molecule cm^{-3}, P_T = 5.0−30.0 Torr, and T = 298 K.

References

[1] Schmitt, G.; Comes, F. J. Photolysis of CH_2I_2 and 1,1-$C_2H_4I_2$ at 300 nm. *J. Photochem.* **1980**, *14*, 107−123.

[2] Howes, N. U. M.; Mir, Z. S.; Blitz, M. A.; Hardman, S.; Lewis, T. R.; Stone, D.; Seakins, P. W. Kinetic Studies of C_1 and C_2 Criegee Intermediates with SO_2 Using Laser Flash Photolysis Coupled with Photoionization Mass Spectrometry and Time Resolved UV Absorption Spectroscopy. *Phys. Chem. Chem. Phys.* **2018**, *20*, 22218−22227.

[3] Luo, P.-L.; Endo, Y.; Lee, Y.-P. Identification and Self-reaction Kinetics of Criegee Intermediates *syn*-CH_3CHOO and CH_2OO via High-resolution Infrared Spectra with a Quantum-cascade Laser. *J. Phys. Chem. Lett.* **2018**, *9*, 4391−4395.

[4] Sheps, L.; Scully, A. M.; Au, K. UV Absorption Probing of the Conformer-Dependent Reactivity of a Criegee Intermediate CH_3CHOO. *Phys. Chem. Chem. Phys.* **2014**, *16*, 26701−26706.

[5] Ting, W.-L.; Chang, C.-H.; Lee, Y.-F.; Matsui, H.; Lee, Y.-P.; Lin, J. J.-M. Detailed Mechanism of the $CH_2I + O_2$ Reaction: Yield and Self-reaction of the Simplest Criegee Intermediate CH_2OO. *J. Chem. Phys.* **2014**, *141*, 104308.

[6] Gravestock, T. J.; Blitz, M. A.; Bloss, W. J.; Heard, D. E. A Multidimensional Study of the Reaction $CH_2I + O_2$: Products and Atmospheric Implications. *ChemPhysChem* **2010**, 11, 3928−3941.
